# The Fragility Index of Randomized Controlled Trials for Preterm Neonates

**DOI:** 10.3389/fped.2022.876366

**Published:** 2022-05-09

**Authors:** Huiyi Li, Zhenyu Liang, Qiong Meng, Xin Huang

**Affiliations:** ^1^Department of Pediatrics, Guangdong Second Provincial General Hospital, Guangzhou, China; ^2^Center for Clinical Epidemiology and Methodology (CCEM), Guangdong Second Provincial General Hospital, Guangzhou, China

**Keywords:** fragility index, robustness, randomized controlled trial, premature, neonate

## Abstract

**Background:**

As a metric to determine the robustness of trial results, the fragility index (FI) is the number indicating how many patients would be required to reverse the significant results. This study aimed to calculate the FI in randomized controlled trials (RCTs) involving premature.

**Methods:**

Trials were included if they had a 1:1 study design, reported statistically significant dichotomous outcomes, and had an explicitly stated sample size or power calculation. The FI was calculated for binary outcomes using Fisher’s exact test, and the FIs of subgroups were compared. Spearman’s correlation was applied to determine correlations between the FI and study characteristics.

**Results:**

Finally, 66 RCTs were included in the analyses. The median FI for these trials was 3.00 (interquartile range [IQR]: 1.00–5.00), with a median fragility quotient of 0.014 (IQR: 0.008–0.028). FI was ≤ 3 in 42 of these 66 RCTs (63.6%), and in 42.4% (28/66) of the studies, the number of patients lost to follow-up was greater than that of the FI. Significant differences were found in the FI among journals (*p* = 0.011). We observed that FI was associated with the sample size, total number of events, and reported *p*-values (*r*_*s*_ = 0.437, 0.495, and −0.857, respectively; all *p* < 0.001).

**Conclusion:**

For RCTs in the premature population, a median of only three events was needed to change from a “non-event” to “event” to render a significant result non-significant, indicating that the significance may hinge on a small number of events.

## Introduction

Preterm birth is a live birth that occurs before 37 completed weeks of pregnancy (1), and the rates range from approximately 5% in some European countries to 18% in some African countries (2). Yearly, an estimated 15 million infants are born preterm, and this number is rising (1, 2). The risk of adverse outcomes in preterm infants rise sharply with decreasing gestational age. These risks have an impact across the neonate’s life course, and previous studies have reported both short- and longer-term conditions associated with preterm birth, including respiratory, infectious, neurocognitive, mental and neurological diseases in childhood and adulthood (3–5). The latest estimates suggest that globally, complications of preterm birth were the leading cause of death in children under 5 years, accounting for approximately 16% of under-five mortality and 35% of deaths among newborns (2, 6). Approximately one million infants die every year due to complications of preterm births (6). Three-quarters of these deaths could be prevented with current cost-effective interventions (1). When preterm labor is inevitable or has taken place, effective interventions to prevent associated complications are actually more significant.

The most reliable methods for measure the impact of interventions and establishing causality come from rigorously conducted and adequately powered randomized controlled trials (RCTs); however, RCTs of interventions for preterm neonates often provide discordant results. Fragility index (FI) is the minimum number of events (in the control or experimental group) that needs to move from “non-event” (not experiencing an endpoint) to “event” (experiencing an endpoint) to render a significant result non-significant (7). A small FI indicates a fragile clinical trial result, whereas a large FI means a robust result hinging on a larger number of patients. The FI is a metric to determine the solidity of statistically significant results of dichotomous outcomes in RCTs (7, 8). To date, no study has evaluated the statistical fragility of RCTs involving preterm neonates. The goal of our study was to measure the robust of clinical trials for premature using the metric of FI, and to describe the associated trial characteristics.

## Materials and Methods

### Identification of Clinical Trials and Data Extraction

We searched PubMed to identify all RCTs on preterm neonates, using the keywords “preterm,” “premature,” “neonat*,” “newborn,” “randomized controlled trial,” “clinical trial,” and “mortality.” The most recent search was performed on December 28, 2021. Studies were included if they were RCTs in the premature population with statistically significant findings for dichotomous primary (or secondary) outcomes, in which there was an explicitly stated sample size or power calculation, with parallel arm study design.

For each trial, the following information was collected: first author, year of publication, title, journal, multicenter, double-blinded, trial registry, observed numbers of events for the control and intervention groups for the outcomes, randomized sample size, and the number of patients lost to follow-up (%).

Two investigators (HL and ZL) independently screened the studies. Any disagreement was resolved through discussion with a third reviewer (XH).

### Statistical Analysis

The FI was calculated in a two-by-two contingency table based on the data used in the original analyses of RCTs (7). The FI was calculated by adding an event from the group with a smaller number of events (and subtracting a non-event from the same group to keep the total number of patients constant) and recalculating the two-sided *p*-value for Fisher’s exact test (7, 8). The FI was the smallest number of added events required to result in *p* ≥ 0.05 (7), which was computed by the online calculator (9) available at https://clincalc.com/Stats/FragilityIndex.aspx. Primary outcome was used to calculate the FI, and if there was no significant dichotomous primary outcome, secondary outcome was used as alternative. For trials reporting multiple significant outcomes, data were analyzed only for the outcome with the smallest FI. Since the trial sample size may alter the FI, the fragility quotient (FQ) was also calculated, which equaled FI divided by the total sample size (10).

Continuous variables were reported as medians with interquartile ranges (IQRs), whereas categorical data were presented as counts with percentages. The Mann–Whitney *U* test was used to compare the median FIs of the two groups, and the Kruskal–Wallis test was performed for three or more groups (11, 12). Spearman’s correlation was applied to analyze the correlations between the FI and different variables. All analyses were conducted using the R software version 3.6.0 (R Development Core Team, Vienna, Austria). Two-sided significance testing was used, and statistical significance was set at *p* < 0.05.

## Results

### Search Results

The literature search identified 1,196 potentially eligible studies. After screening titles and abstracts, 757 studies were excluded. On full-text review, a further 373 publications were excluded for reasons including unavailability of full text, not RCTs on preterm neonates, no explicitly stated sample size or power calculation, no significant dichotomous outcome reported, not 1:1 two-arm design, and *post hoc* or subgroup analysis. Ultimately, 66 RCTs were included in this study (Supplementary Figure 1).

### Trial Characteristics and Fragility Index

The included trials and their corresponding FIs are listed in Supplementary Table 1. The median sample size in the analyzed RCTs was 149.5 (IQR: 80-367.5), and the median number of intervention events was 38 (IQR: 23–95.25). Table 1 lists the characteristics of the included trials and the FI by subgroup. Thirty-four (51.5%) trials were published after 2010. Most RCTs were multicenter-designed (56.1%) and conducted on the intention-to-treat principle (59.1%). For 41 trials (62.1%), the primary outcome was used for FI calculation.

**TABLE 1:** Characteristics of included trials and fragility index by subgroup.

Characteristics	Description, *n* (%)	Median FI (IQR)	*p*
Number of patients lost to follow-up (NPLFU)			0.495
NPLFU > fragility index	28 (42.4)	3.00(1.00−5.00)	
NPLFU ≤ fragility index	38 (57.6)	2.50(1.00−5.00)	
Year of publication			0.245
Prior to 2000	14 (21.2)	3.00(1.00−3.50)	
2000-2011	18 (27.3)	3.50(1.00−5.25)	
2011-present	34 (51.5)	2.50(1.00−5.00)	
Multicenter			0.116
Yes	37 (56.1)	3.00(2.00−5.00)	
No or not sure	29 (43.9)	2.00(1.00−3.50)	
Double-blinded			0.786
Yes	22 (33.3)	3.00(1.00−4.25)	
No or not sure	44 (66.7)	3.00(1.00−5.00)	
RCT registration			0.065
Yes	30 (45.5)	3.50(2.00−6.25)	
No or not reported	36 (54.5)	2.00(1.00−3.75)	
Intention-to-treat analysis			0.493
Yes	39 (59.1)	3.00(2.00−5.00)	
No	27 (40.9)	2.00(1.00−4.00)	
Outcome used for analysis			0.172
Primary outcome	41 (62.1)	3.00(1.00−5.00)	
Secondary outcome	25 (37.9)	2.00(1.00−3.50)	
Journal			**0.011**
New England Journal of Medicine	12 (18.2)	5.00(3.00−10.25)	
Journal of Pediatrics	7 (10.6)	2.00(1.00−3.00)	
Neonatology	7 (10.6)	3.00(2.00−7.00)	
Pediatrics	7 (10.6)	5.00(1.00−5.00)	
JAMA	3 (4.5)	4.00(*na*)	
Other	30 (45.5)	1.50(1.00−3.00)	

*The significant result was in bold.*

**FIGURE 1:**
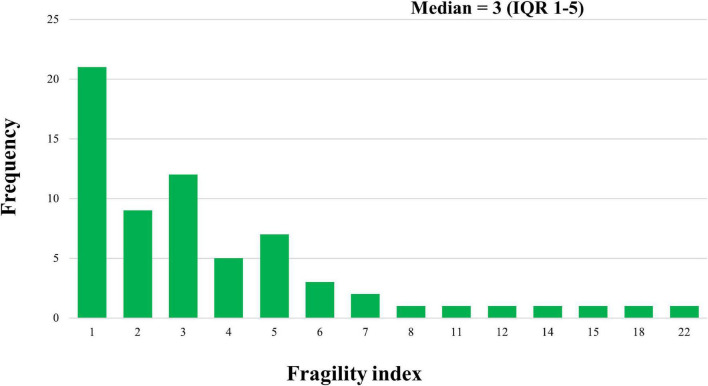
Distribution of Fragility index across randomized controlled trials of preterm neonates. IQR: interquartile range.

The median FI for eligible trials was 3.00 (IQR: 1.00–5.00), ranging from 0 to 22, with a median FQ of 0.014 (IQR: 0.008–0.028). Twenty-one trials (31.8%) had a FI of 1, whereas FI was ≤ 3 in 42 of the trials (63.6%; Figure 1). In 42.4% (28/66) of the studies, the number of patients lost to follow-up was greater than that of the FI. Significant differences in the FI among journals were found (*p* = 0.011, Table 1): FI was 5.00 (IQR: 3.00–10.25) for the *New England Journal of Medicine*, 2.00 (IQR: 1.00–3.00) for the *Journal of Pediatrics*, 3.00 (IQR: 2.00–7.00) for *Neonatology*, and 5.00 (IQR: 1.00–5.00) for *Pediatrics*. However, the results of the trials based on other trial characteristics (e.g., multicenter, double-blind, trial registration) did not differ in the degree of robustness (all *p* > 0.05).

### Correlation Between the FI and Trial Characteristics

**FIGURE 2:**
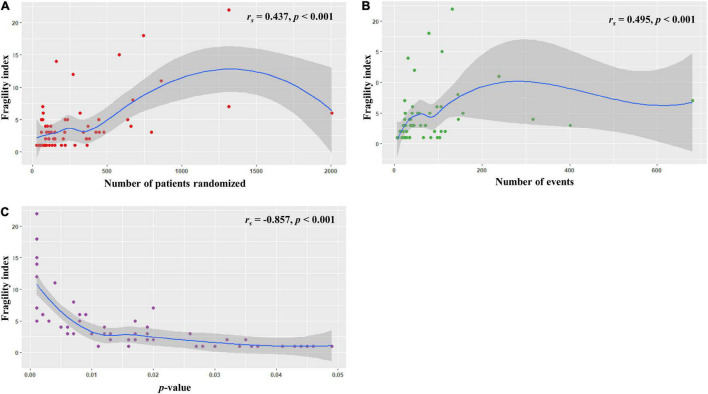
Relationships between the fragility index and **(A)** sample size, **(B)** total number of events, and **(C)** *p* value.

We found that sample size (defined by the total number of randomized participants) and total number of events were positively correlated with the FI (*r*_*s*_ = 0.437 and 0.495, respectively, all *p* < 0.001; Figures 2A,B). In contrast, a strong negative correlation was observed between the FI and reported *p*-values (*r_*s*_* = −0.857, *p* < 0.001; Figure 2C).

## Discussion

Our investigation demonstrated the fragility of the trial outcomes from 66 RCTs on premature neonates. The results showed that a median of three event alterations would be needed to nullify the statistical significance of trial outcomes. Forty-two of the RCTs (63.6%) had a FI ≤ 3. In 42.4% of the trials, the number of patients lost to follow-up was greater than that of the FI. Significant differences were observed in the FI among journals. Additionally, we found that FI was significantly associated with the sample size, total number of events, and reported *p*-values.

Fragility index is defined as the minimum number of patients that need to be transposed from “non-event” to “event” to change the findings from significant to non-significant. A small FI manifests that a subsequent trial may overturn the results of the current trial. The median FI from clinical trials of spine surgery (13), anticancer medicines (14), and ophthalmology (15) have all been 3 or less. Medical literature in other areas have shown similar values. For example, in hand surgery, the median FI was 3 (range 0–26) (16), and in critical care trials, it was 2 (IQR 1–3.5) (17). There were also studies with a large FI, such as diabetes (9) and heart failure (18), in which the RCTs were retrieved from the treatment guidelines. The large FIs of the studies suggested that the clinical guidelines were based on RCTs with robust results, highlighting the solidity of the guidelines. In this study, clinical trials were retrieved from a database search, similar to most other similar studies mentioned above, and our study aimed to evaluate the statistical fragility of RCTs in the premature population. Our study showed that a median of three event alterations would be needed to reverse the statistical significance of trial outcomes for premature.

We also observed that FI was associated with the sample size, total number of events, and reported *p*-values (all *p* < 0.001), which were consistent with previous studies (12, 19). In our study, significant differences were also found in the FI among journals. Especially, median FI was 5.00 (IQR: 3.00–10.25) for the *New England Journal of Medicine* (impact factor = 90.59), and 2.00 (IQR: 1.00–3.00) for the *Journal of Pediatrics* (impact factor = 4.41). This indicated that higher quality journals may publish more solid data and should be thoroughly studied by pediatrician (20). At this point, our study partly verified this widely accepted viewpoint within scientific researches in a quantitative way. Sufficient sample size underlies the statistical power required for the clinical trials. Thus, RCTs published in higher quality journals tended to have, on average, larger sample sizes, and ultimately, also higher incidence of “events” (15). Positive correlation was observed between FI and impact factor of the journal of publication in previous literatures (11, 12). These together may help comprehend the differences in the FI among journals.

The use of the *p*-value has been criticized in recent years (21). Relying on a fixed *p*-value level has been considered as one of the potential reasons of the low replication rate in scientific research (12). The FI may be a tangible metric that can be used alongside *p*-values and effect sizes to provide an intuitive measure of the robustness of trial results (12). Compared with the conventional *p*-value, which needs to be understood from a statistical point of view, the FI is a plain number. The FI may be more intelligible for clinicians who lack statistical knowledge in most cases, resulting in more informed, evidence-based clinical decisions.

As a relative measure of fragility, the FQ was calculated by referencing the FI to the trial sample size (10). We reported FQ as a supplement to the FI, and a median FQ of 0.014 was found (IQR: 0.008–0.028). This meant that 14 per 1,000 patients with a non-event altered to an event would result in a loss of significance. Similar to the FI, a smaller FQ means a more fragile and less statistically robust RCT result. In particular, for trials with the same or similar FI, the FQ will help compare the fragility of the trials.

Notably, in 42.4% of the trials, the number of patients lost to follow-up was greater than that of the FI. In this condition, the patients lost to follow-up may have provided sufficient data to change the reported statistical significance of the results and ultimately influence the robustness of a trial (11). Therefore, these results should be interpreted with caution.

To the best of our knowledge, this is the first study to assess fragility in premature clinical trials. However, there were still several limitations. First, PubMed comprises more than 33 million citations for biomedical literature from MEDLINE, life science journals, and online books, and almost all the publications in PMC are included in PubMed. However, we only searched PubMed for eligible literatures, which might lead to missing some studies. Second, according to related definitions, only two-arm clinical trials with significant dichotomous outcomes were included in the analyses. Our results may lack data from well-conducted clinical trials that report negative and/or continuous outcomes. A method for calculating the FI of continuous outcomes has been introduced recently (22). However, there is still no defined interpretation of continuous FI. In future studies, this technique may be applied to premature areas and then provide additional information. Third, there has been no threshold value to declare a result “fragile” or “robust.” In most cases, the FI was used as a relative measure to compare the fragility of two independent clinical trials (or the median FI for two RCT clusters). Forth, as sample size was powered for the primary endpoint, the FI calculated using secondary endpoints must be viewed with caution. Moreover, there are no conventional methods to evaluate the quality of this methods, which may compromise the validity of our findings owing to the lack of assessment of evidence strength.

## Conclusion

Our study showed that, for premature trials, a median of only three events was needed to alter from a “non-event” to “event” to render a significant result non-significant. Although the results of RCTs in the premature population may be statistically significant based on the *p*-value (≤ 0.05), the significance may rely on a small number of events. FI, as a supplementary metric used alongside *p*-values and effect sizes, represents an important aid to the clinician’s interpretation of trial results.

## Data Availability Statement

The original contributions presented in the study are included in the article/Supplementary Material, further inquiries can be directed to the corresponding author/s.

## Ethics Statement

Ethical review and approval was not required for the study on human participants in accordance with the local legislation and institutional requirements. Written informed consent for participation was not required for this study in accordance with the national legislation and the institutional requirements.

## Author Contributions

HL and XH conceived and designed the study, and drafted the manuscript. ZL, HL, and QM contributed to data acquisition and analysis. All authors contributed to data analysis, drafting or revising the manuscript, gave final approval of the version to be published, and agreed to be accountable for all aspects of this work, participated in the interpretation of the results and critically revised the manuscript for important intellectual content.

## Conflict of Interest

The authors declare that the research was conducted in the absence of any commercial or financial relationships that could be construed as a potential conflict of interest.

## Publisher’s Note

All claims expressed in this article are solely those of the authors and do not necessarily represent those of their affiliated organizations, or those of the publisher, the editors and the reviewers. Any product that may be evaluated in this article, or claim that may be made by its manufacturer, is not guaranteed or endorsed by the publisher.
