# Supplementary figures and images for: The Fragility Index of Randomized Controlled Trials for Preterm Neonates
Source: Front Pediatr. 2022 May 9;10:876366. doi: 10.3389/fped.2022.876366 (PMC9124941; doi:10.3389/fped.2022.876366)

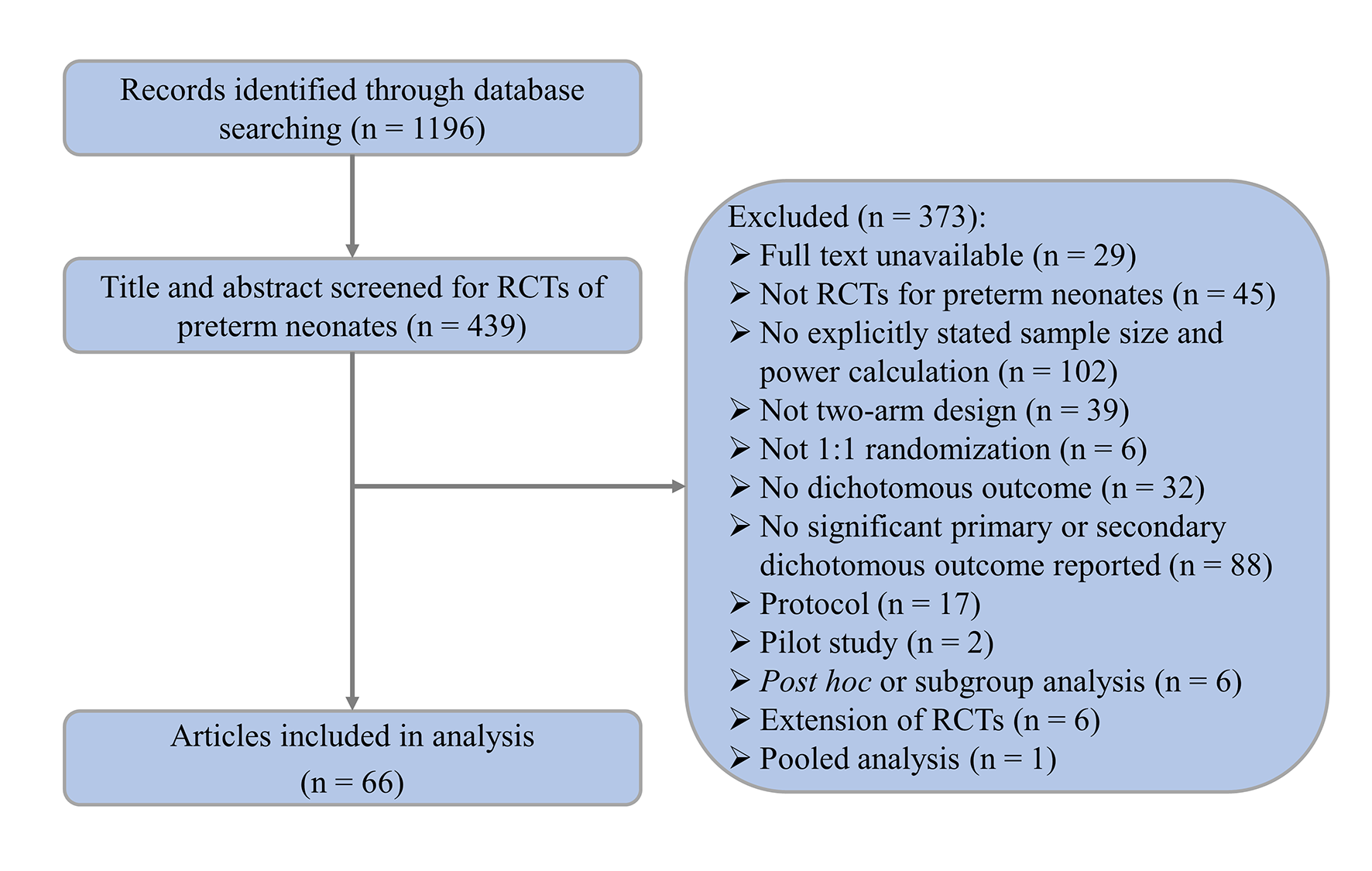

Supplement: Supplementary file 2 [file Image_1.TIF]
